# Traumatic Dislocation of the First Carpometacarpal Joint in a Skier: A Case Report and Review of the Literature

**DOI:** 10.7759/cureus.98943

**Published:** 2025-12-10

**Authors:** Ahmed Al-Sadek, Georgi Luchev, Lyubomir Gaydarski, Georgi P Georgiev

**Affiliations:** 1 Orthopaedics and Traumatology, University Hospital Queen Giovanna - ISUL, Sofia, BGR; 2 Anatomy, Histology and Embryology, Medical University of Sofia, Sofia, BGR

**Keywords:** case report, conservative management, dorsoradial ligament, first carpometacarpal joint dislocation, ligament reconstruction, skiing injury, thumb dislocation

## Abstract

Traumatic dislocation of the first carpometacarpal joint is a rare orthopedic injury. It is typically associated with high-energy trauma in young males but can also occur in sporting accidents such as skiing. We present the case of a 30-year-old female skier who sustained a dorsal dislocation of the first CMC joint while skiing. The injury occurred due to a mechanical fall while gripping a ski pole, a mechanism distinct from the typical axial loading seen in other traumas. The dislocation was managed with closed reduction and immobilization in a thumb spica cast. Although standard protocols typically recommend four to six weeks of immobilization to ensure ligamentous healing, the patient independently removed the cast at 21 days post injury. Despite this early removal, clinical and radiographic follow-up at 30 days demonstrated a stable, concentric joint with good functional outcomes and minimal pain. In conclusion, early recognition and anatomical reduction are paramount for the successful management of first carpometacarpal dislocations. While four to six weeks of immobilization remains the standard of care to prevent chronic instability, this case demonstrates that satisfactory stability and function can be achieved with shorter immobilization periods in stable injuries.

## Introduction

The first carpometacarpal (CMC) joint is a biconcave saddle joint that permits a wide range of motion, including flexion, extension, abduction, adduction, and opposition. This mobility, which is critical for human prehension, comes at the cost of intrinsic osseous stability. Stability is primarily provided by the complex capsuloligamentous structures, most notably the dorsoradial ligament (DRL) and the anterior oblique ligament (AOL), also known as the "beak" ligament [1-3]. The joint surfaces are reciprocally interlocking, but this bony congruity is insufficient to maintain stability under high physiological loads without intact ligamentous support [4,5].

Dislocations of the first CMC joint are uncommon, typically resulting from high-energy axial loading on a flexed thumb [6,7]. They are distinct from the more common Bennett's or Rolando's fracture-dislocations in that they lack a significant intra-articular fracture fragment, although small rim avulsions may occasionally be present [8]. Due to the rarity of the injury, optimal management remains a subject of debate. While some authors advocate for conservative management with casting for stable reductions, citing successful outcomes in acute cases [1,2], others argue that the high rate of recurrent instability, reported to be as high as 60% in some series, necessitates percutaneous pinning or open ligament repair to prevent chronic pain, instability, and post-traumatic osteoarthritis [9].

This report presents a case of a skiing-related dislocation in a 30-year-old female skier that was managed conservatively with early mobilization. We also provide a comprehensive review of the current literature to clarify the pathophysiology, anatomical controversies regarding joint stability, and optimal treatment algorithms for this injury.

## Case presentation

**Figure 1:**
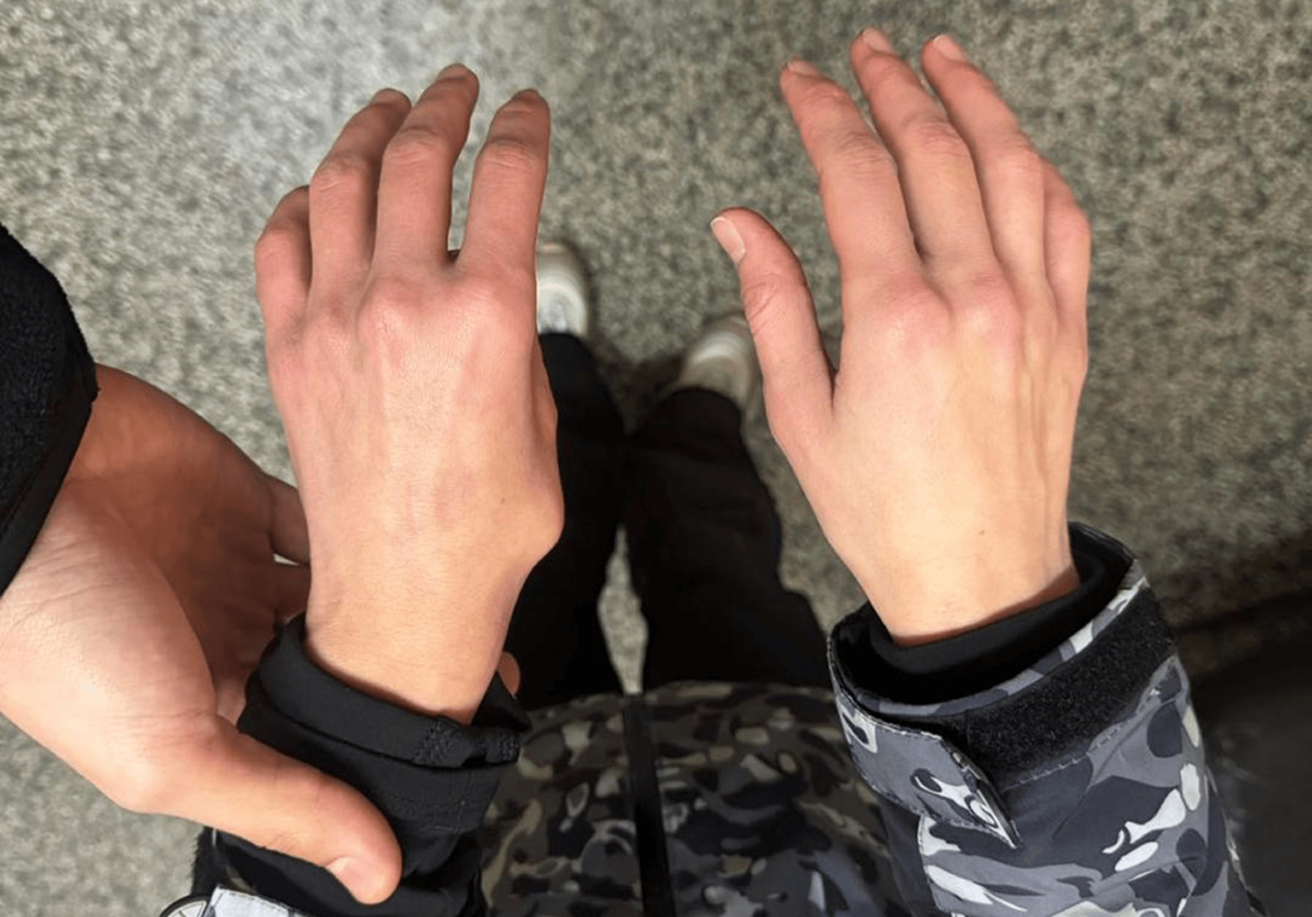
Post-injury photograph showing a palpable bony prominence at the dorsum of the thumb base.

A 30-year-old female skier presented with acute pain, swelling, and deformity at the base of her right thumb. The injury occurred while skiing; she reported a mechanical fall while gripping the ski pole, which forced her thumb into abduction and extension. She immediately noticed a deformity and was unable to move the thumb. On physical examination, there was a palpable bony prominence at the dorsum of the thumb base (Figure 1), corresponding to the displaced metacarpal base, and a distinct inability to oppose the thumb.

**Figure 2:**
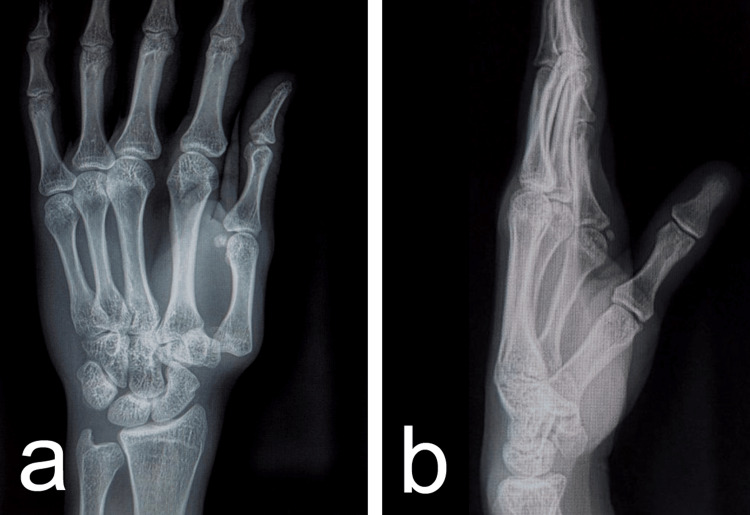
Diagnostic anteroposterior (a) and lateral (b) radiographs showing dorsal dislocation of the first carpometacarpal joint.

There was diffuse tenderness over the first CMC joint and thenar eminence. The neurovascular status of the digit was intact, with normal two-point discrimination and capillary refill. Standard anteroposterior and lateral radiographs of the hand revealed a pure dorsal dislocation of the first metacarpal relative to the trapezium (Figure 2). The joint space was completely disrupted, and the metacarpal base was displaced proximally and dorsally. No associated fractures, such as Bennett's or Rolando's fractures, were identified.

Closed reduction was performed under local anesthesia (hematoma block). The reduction maneuver involved longitudinal traction to disengage the metacarpal base, followed by direct volar pressure on the metacarpal base while maintaining the thumb in abduction. A palpable "clunk" was felt upon reduction. Stability was assessed under fluoroscopy; the joint was stable through a functional range of motion, and no gross instability was noted upon mild stress testing. The hand was immobilized in a thumb spica cast to maintain the reduction.

**Figure 3:**
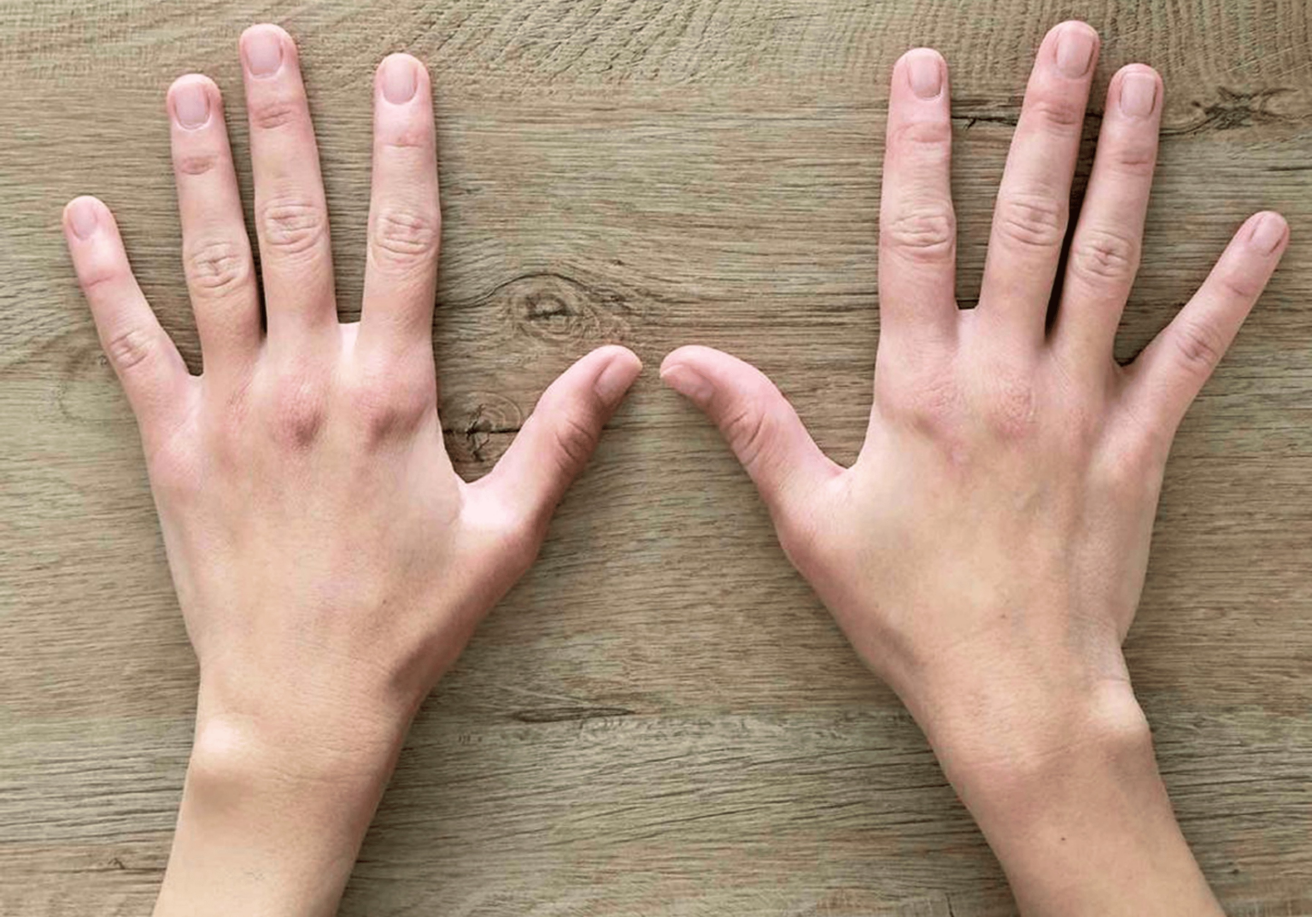
Photograph at the 30-day follow-up presents no deformation at the dorsum of the thumb base.

The patient was instructed to maintain the cast for a standard period of four to six weeks to allow for ligamentous healing. However, the patient removed the cast independently on post-injury day 21. Despite this early removal of immobilization, she presented for follow-up at 30 days, reporting minimal pain and good function. Clinical examination at the 30-day follow-up revealed a stable first CMC joint with no tenderness (Figure 3).

**Figure 4:**
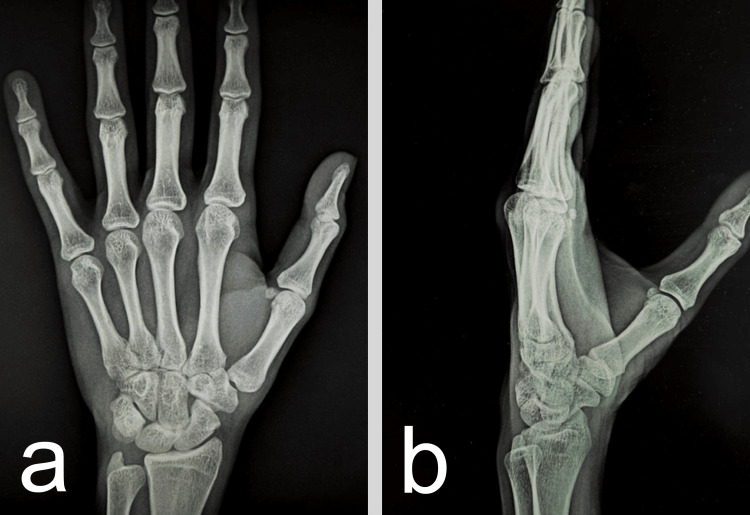
Follow-up anteroposterior (a) and lateral (b) radiographs showing no signs of subluxation or joint space narrowing

Follow-up radiographs confirmed maintenance of concentric reduction with no signs of subluxation or joint space narrowing (Figure 4).

The patient was advised to avoid heavy gripping and contact sports for an additional four weeks to ensure complete ligamentous healing and prevent late instability. At the three-month follow-up, the patient reported a QuickDASH (Quick Disabilities of the Arm, Shoulder, and Hand) score of 1 [10], indicating excellent upper limb function and a return to pre-injury activities.

## Discussion

Isolated dislocation of the thumb CMC joint represents less than 1% of all hand injuries [6,11]. A 2022 systematic review by Court et al. found that the injury predominantly affects young male patients (86.1% of reported cases), with a mean age of approximately 31 years [11]. Our case involves a 30-year-old female skier who falls outside the typical gender demographic but aligns with the age distribution commonly seen in these injuries. The rarity of the injury often leads to delayed diagnosis or misdiagnosis as a simple sprain, emphasizing the need for careful clinical and radiographic evaluation.

The injury typically occurs due to axial compression transmitted through a partially flexed thumb metacarpal [12]. Common mechanisms include falls onto an outstretched hand, bicycle/motorcycle accidents, and sports-related trauma [1,13,14]. Kraus and Weaver highlighted a specific mechanism in skiers known as the "ski pole" mechanism [2]. In this scenario, the ski pole acts as a fulcrum during a fall. As the skier falls, the strap or handle forces the thumb into hyperextension and abduction. This levering action drives the metacarpal base dorsally, resulting in dislocation. This mechanism is consistent with our patient's history and differs slightly from the direct axial load seen in boxing or motorcycle accidents. Understanding this mechanism is crucial for physicians treating winter sports injuries, as the force vector may create different patterns of soft tissue injury compared to direct impact trauma.

The stability of the first CMC joint has been a subject of significant anatomical debate. Historically, the AOL, or "beak ligament," was considered the primary stabilizer against dorsal dislocation [15]. This belief drove the development of the classic Eaton-Littler reconstruction, which focuses on recreating the volar beak ligament using a flexor carpi radialis (FCR) tendon graft. However, more recent biomechanical studies, notably by Strauch et al. [4], have challenged this view. They demonstrated that the DRL complex is the primary restraint to dorsal dislocation. Their cadaveric sectioning studies showed that isolated sectioning of the AOL resulted in volar instability but not dorsal dislocation, whereas sectioning of the DRL resulted in significant dorsal subluxation. Intraoperative findings in multiple case reports confirm that the dorsal capsule and DRL are often torn or avulsed. At the same time, the volar ligaments remain intact, often peeled off the metacarpal periosteum but structurally continuous [9,16]. This shift in understanding has profound implications for surgical management, suggesting that repair or reconstruction should target the dorsal complex rather than the volar structures. The key ligaments involved are summarized in Table 1.

**Table 1:** Anatomical stabilizers of the first carpometacarpal joint.

Ligament	Primary Function	Role in Dorsal Dislocation	Supporting Evidence
Dorsoradial (DRL)	Primary restraint to dorsal subluxation.	Essential lesion; rupture permits dorsal displacement.	Strauch et al. [4], Fotiadis et al. [9]
Anterior Oblique (AOL)	Restraint to volar subluxation ("Beak" ligament).	Often peeled off the metacarpal but remains structurally intact.	Eaton and Littler [15]
Posterior Oblique (POL)	Secondary stabilizer.	May be attenuated or torn during high-energy trauma.	Bettinger et al. [5]
Intermetacarpal (IML)	Connects 1st and 2nd metacarpal bases.	Prevents radial translation of the first ray.	Court et al. [11]

The treatment is dictated by the stability of the joint following reduction. There is no universal consensus, but the literature supports a stepped-care approach. Table 2 summarizes the key findings from the literature regarding different treatment modalities.

**Table 2:** Summary of selected literature on first carpometacarpal joint dislocation management. * total patients included in the systematic review. FCR: flexor carpi radialis; DRL: dorsoradial ligament

Study (Year)	Study Type	Treatment Modality	Key Findings / Outcomes
Bosmans et al. [3] (2008)	Case Series (N=2)	Closed Reduction + Cast	Excellent functional outcome at 3 years. Concluded surgery is not always mandatory for acute, stable cases.
Fotiadis et al. [9] (2010)	Case Report	Open Repair (Suture Anchor)	Full return to sports. Identified the DRL as the key structure needing repair.
Kraus and Weaver [2] (2014)	Case Report	Closed Reduction + Splint	Stable reduction in a skier. Emphasized the importance of early diagnosis to allow for conservative care.
Lahiji et al. [12] (2015)	Case Series (N=6)	Mixed (Cast & Surgery)	5/6 required surgery; 1 treated with cast had a good result. Highlighted high rate of instability necessitating surgery.
Slocum and Lui [1] (2019)	Case Report	Closed Reduction + Splint	Asymptomatic at 2 years. Emphasized casting if the joint is stable post-reduction.
Court et al. [11] (2022)	Systematic Review (N=37*)	Surgical Reconstruction	96% of reconstructions used FCR tendon. Confirmed DRL is the primary focus of modern reconstruction.
Punekar et al. [17] (2025)	Case Report	FCR Reconstruction	Excellent score (Kapandji 10) for chronic instability using FCR tendon transfer.

Table 3 provides a direct comparison of the three main treatment strategies discussed in the literature. This summary highlights the relative advantages, limitations, and clinical considerations associated with each approach, enabling a clearer understanding of how they differ in terms of stability, soft-tissue preservation, complication rates, and postoperative outcomes. 

**Table 3:** Comparison of treatment modalities for first carpometacarpal joint dislocation. CRPP: closed reduction percutaneous pinning

Modality	Indication	Advantages	Disadvantages
Closed Reduction + Casting	Acute, stable reduction; concentric joint on X-ray.	Non-invasive, lower cost, avoids surgical risks.	Higher risk of re-dislocation, potential for missed instability.
Percutaneous Pinning (CRPP)	Unstable reduction; high risk of re-dislocation.	Provides rigid stabilization, minimally invasive.	Pin tract infection, pin migration, does not repair ligaments directly.
Ligament Reconstruction	Chronic instability, irreducible dislocation, high-demand athletes.	Restores anatomical stability, addresses ligament pathology directly.	Invasive, longer recovery, risks of surgery (nerve injury, stiffness).

The most notable aspect of this case is the successful outcome despite the patient's removal of the cast after 21 days. This stability at 30 days suggests that in cases where the reduction is concentric and the dorsal soft tissue envelope is approximated, ligamentous healing may occur more rapidly than traditionally thought. This aligns with the findings of Bosmans et al., who argued that if the joint is congruent post-reduction, extensive surgical reconstruction may not be necessary for acute cases [3]. The congruency of the joint likely allows for healing of the dorsal structures in an approximated position, restoring the tension band effect of the DRL.

However, this does not imply that early mobilization is risk-free. Missed diagnosis remains a significant risk; swelling often masks the "step-off" deformity [2]. Watt and Hooper reported high rates of instability with casting alone, particularly in delayed presentations [18]. Therefore, conservative treatment necessitates rigorous radiographic follow-up. If conservative management is chosen, we recommend weekly radiographs for the first three weeks to detect any subluxation early. Future studies should utilize standardized metrics, such as the DASH (Disabilities of the Arm, Shoulder, and Hand) score and Kapandji thumb opposition score, as recommended by Court et al., to better quantify outcomes between conservative and surgical groups [11]. Such data would help stratify patients who are ideal candidates for conservative care versus those who require immediate stabilization.

## Conclusions

Traumatic first CMC dislocation is a rare injury that requires a high index of suspicion, especially in skiers, where the "ski pole" mechanism is prevalent. While four to six weeks of immobilization is the standard of care, this case demonstrates that satisfactory stability and function can be achieved with shorter immobilization periods (three weeks) in select, stable injuries. Conservative management remains a viable first-line treatment, provided the reduction is anatomical and monitored closely for recurrence. Surgeons should maintain a low threshold for operative intervention if instability persists, but avoid unnecessary surgery in compliant patients with stable reductions.
